# Human Colon-Derived Soluble Factors Modulate Gut Microbiota Composition

**DOI:** 10.3389/fonc.2015.00086

**Published:** 2015-04-13

**Authors:** Arancha Hevia, David Bernardo, Enrique Montalvillo, Hafid O. Al-Hassi, Luis Fernández-Salazar, Jose A. Garrote, Christian Milani, Marco Ventura, Eduardo Arranz, Stella C. Knight, Abelardo Margolles, Borja Sánchez

**Affiliations:** ^1^Department of Microbiology and Biochemistry of Dairy Products, Instituto de Productos Lácteos de Asturias – Consejo Superior de Investigaciones Científicas (IPLA-CSIC), Villaviciosa, Spain; ^2^Antigen Presentation Research Group, Imperial College London, Harrow, UK; ^3^Gastroenterology Unit, Hospital Universitario de La Princesa and Instituto de Investigación Sanitaria Princesa (IIS-IP), Centro de Investigación Biomédica en Red de Enfermedades Hepáticas y Digestivas (CIBEREHD), Madrid, Spain; ^4^Mucosal Immunology Laboratory, Instituto de Biología y Genética Molecular (IBGM), University of Valladolid-CSIC, Valladolid, Spain; ^5^Gastroenterology Service, Hospital Clinico Universitario de Valladolid, Valladolid, Spain; ^6^Clinical Laboratory Service, Department of Genetics and Molecular Biology, Hospital Universitario Rio Hortega, Valladolid, Spain; ^7^Laboratory of Probiogenomics, Department of Life Sciences, University of Parma, Parma, Italy; ^8^Nutrition and Bromatology Group, Department of Analytical and Food Chemistry, Food Science and Technology Faculty, University of Vigo, Ourense, Spain

**Keywords:** host–microbiota interaction, molecular crosstalk, soluble mediators, microbial modulation, metagenomics

## Abstract

The commensal microbiota modulates immunological and metabolic aspects of the intestinal mucosa contributing to development of human gut diseases including inflammatory bowel disease. The host/microbiota interaction often referred to as a crosstalk, mainly focuses on the effect of the microbiota on the host neglecting effects that the host could elicit on the commensals. Colonic microenvironments from three human healthy controls (obtained from the proximal and distal colon, both in resting conditions and after immune – IL-15- and microbiota – LPS-*in vitro* challenges) were used to condition a stable fecal population. Subsequent 16S rRNA gene-based analyses were performed to study the effect induced by the host on the microbiota composition and function. Non-supervised principal component analysis (PCA) showed that all microbiotas, which had been conditioned with colonic microenvironments clustered together in terms of relative microbial composition, suggesting that soluble factors were modulating a stable fecal population independently from the treatment or the origin. Our findings confirmed that the host intestinal microenvironment has the capacity to modulate the gut microbiota composition via yet unidentified soluble factors. These findings indicate that an appropriate understanding of the factors of the host mucosal microenvironment affecting microbiota composition and function could improve therapeutic manipulation of the microbiota composition.

## Introduction

The immune system of the gastrointestinal tract (GIT) is exposed to a large amount of foreign but harmless antigens typically derived from nutrients and commensal bacteria but sometimes deleterious when derived from infectious bacteria or viruses. Nevertheless, the GIT immune system is effective in discriminating between maintaining immune tolerance against diet and/or commensal derived antigens, and initiating immune responses against harmful invading pathogens (1). IL-15 is one of the cytokine of the innate immune response, regulating both T and natural killer (NK) cell activation and proliferation (2). The commensal microbiota plays a central role in modulating the outcome of immune responses in the GIT keeping immune homeostasis in health (3). Indeed, germ-free animals have an immature immune system and can develop inflammation, which is reversed once the microbiota is conventionalized (4). The commensal microbiota modulates several aspects of the host including the physiology and/or its nutritional status. The microbiota is also related to several diseases affecting the gut, like in inflammatory bowel diseases (IBD), and also influences diseases in distant organs (5–8). On turn, chronic gut inflammation such as happens in IBD is a risk factor for colorectal cancer. This is apparently a consequence of a high and persistent inflammation at the mucosa levels (9).

Gastrointestinal tract microbiota modulation is a promising area of research aiming at an impact in the clinics for patients suffering from GIT diseases, such as IBD. Traditional approaches included the use of antibiotics and/or pre/pro/synbiotic combinations which, although effective to some extent, usually have short-term success (10). Novel approaches are trying to modulate the abnormal microbiota composition in different ways, such as fecal transplants, which have been effective in treating recurrent *Clostridium difficile* infections and other extra-intestinal diseases (11–13). However, there is variability in the results as the treatments are not effective in all patients, partially due to the stability of the human microbiota (14, 15). Indeed, the possibility exists that the host exerts some selective pressure on the microbiota modulating its composition.

The GIT human microbiota is not stable as it increases its content [up to 10^12^ bacteria per gram of colon content (16)], and changes its composition and metabolism through the GIT tract, likely revealing different adaptations to different GIT sections (17–19). The host–microbiota crosstalk is mediated, at least partially, through bacteria-derived soluble factors and not necessarily via direct cell interaction (20–22) and the microbiota composition can indeed be altered during inflammation (23). Nevertheless, there are few studies regarding the way in which the local gut microenvironment influences the microbiota in terms of composition and functionality.

In the human large bowel, the proximal (right) and distal (left) sections have different embryological origin, blood supply/vascularization, lymphatic drainage, and enzymatic activities (24, 25). Epithelial cells from these compartments have different genetic and epigenetic profiles coupled with different antibody secreting cells; thus, immune differences between the proximal and the distal colon could provide different niches for commensal microorganisms (26–28). Besides, similar to how different patients have different immune response thresholds to external challenges, the proximal and the distal colon may also have different response thresholds to external immune challenges (2, 23). Therefore, we undertook studies of host–microbiota crosstalk in proximal and distal areas of the colonic both in resting conditions and after immune challenges, which could disrupt immune homeostasis affecting the local microenvironment.

Here, we hypothesize that the host can modulate the microbiota composition and metabolic activities. Colonic microenvironments from the human proximal and distal colon (both in resting conditions and after immune challenges) were, therefore, used to condition a stable fecal slurry population. Our results proved that the host exerted a selectively pressure on the microbiota composition via host-derived soluble factors. Main results are discussed below.

## Materials and Methods

### Ethical Statement

The study was approved by the Bioethics Committee from Hospital Clínico Universitario from Valladolid, Spain in compliance with the Declaration of Helsinki. All patients provided informed consent on entry to the study.

### Host factors

#### Biological samples

Colonic biopsies were obtained at colonoscopy from three healthy female controls (ages 40, 57, and 66 years old) who had been referred for colorectal cancer screening and were macroscopically and histologically normal.

Paired samples were collected from the distal (left) and proximal (right) human colon (total of four biopsies from each area) in ice chilled PBS and processed within an hour. One biopsy from each compartment (proximal/distal) was used to assess the profile of intraepithelial lymphocytes (IEL) as described below while the other three biopsies were cultured in 1 ml of complete medium [Dutch modified RPMI 1640 (Sigma-Aldrich, Dorset, UK) containing 2 mM l-glutamine (Sigma-Aldrich) and 10% fetal calf serum (TCS cellworks, Buckingham, UK)] for 24 h in 12 well culture dishes and in the absence of antibiotics (1 biopsy/well) (37°C, 5% CO_2_) in basal conditions or challenged with IL-15 (50 ng/ml, R&D) or LPS (*Escherichia coli*; 10 ng/ml Sigma). After 24 h, media from biopsy culture were centrifuged, filtered (0.2 μm), and cell/bacterial-free supernatants collected and preserved at −80°C. Negative controls included parallel processing (including 24 h incubation in culture dishes within the incubator and subsequent centrifugation and cryopreservation) of complete medium, which had not been conditioned with colonic samples.

#### Intraepithelial lymphocytes

Colonic biopsies were incubated for 1 h under gentle agitation with 1 mM dithiothreitol (DTT) (Sigma-Aldrich) and 1 mM ethylenediaminetetraacetic acid (EDTA) (Sigma-Aldrich) in complete medium after which IEL were released into the medium and collected by centrifugation, washed twice in PBS (Lonza, Braine-l’Alleud, Belgium), and stained with fluorochrome-conjugated antibodies.

#### Antibody staining and flow cytometry acquisition

For IEL subset characterization, monoclonal antibodies with the following specificities and conjugations were used: CD45-PE-Cy7 (HI30), CD103-FITC (Ber-ACT8), CD3-APC (HIT3a), and Tγδ-PE (B1) were purchased from Becton Dickinson while CD4-FITC (13B8.2) and CD8-PE (B9.11) were purchased from Beckman Coulter. Cells were labeled in PBS containing 1 mM EDTA and 0.02% sodium azide (FACS buffer). Labeling was performed on ice and in the dark for 20′. Cells were washed twice in FACS buffer, fixed with 1% paraformaldehyde in 0.85% saline, and stored at 4°C prior to acquisition on a Beckman Coulter FC500 flow cytometer (within 48 h). Appropriate isotype-matched control antibodies were purchased from the same manufacturers. IEL were identified as CD45^+^CD103^+^ and were further characterized as T-cells (CD3^+^) [both classical T-cells (CD3^+^TCRγδ^−^) and Tγδ cells (CD3^+^TCRγδ^+^)] or NK-like cells (CD3^−^). Within T-cells CD4^+^ and CD8^+^subsets were further identified.

#### Culture supernatants

Cell-free culture supernatants were analyzed by using a Flow Cytomix Multiple Analyte Detection (EBioscience) on a BD FACSCanto II flow cytometer (BD) following the manufacturer’s instructions, for the concentration of interferon (IFN) γ [detection limit (D.L.) 0.25 pg/ml]; interleukin (IL)-1β (D.L. 1.80 pg/ml); IL-2 (D.L. 0.35 pg/ml); IL-4 (D.L. 1.22 pg/ml); IL-5 (D.L. 0.76 pg/ml); IL-6 (D.L. 4.10 pg/ml); IL-9 (D.L. 1.17 pg/ml); IL-10 (D.L. 2.88 pg/ml); IL-12p70 (D.L. 0.1 pg/ml); IL-13 (D.L. 0.43 pg/ml); IL-17A (D.L. 0.89 pg/ml); IL-22 (D.L. 3.98 pg/ml); IL-23 (D.L. 29.14 pg/ml); IL-27 (D.L. 0.79 pg/ml); leptin (D.L. 34.95 pg/ml), and tumor necrosis factor α (TNF-α) (D.L. 3.10 pg/ml). IgA content from each compartment was determined with radial immunodiffusion kit (Kit IgA RID-ML, Binding Site, UK, D.L 8.5–85 mg/l) following manufacturer’s instructions. All values below D.L. were reported as being equal to that.

### Microbiota experiments

#### Basal media

Cell-free culture supernatants from the proximal and distal colon, both in resting conditions (basal) and after immune challenges (LPS and IL-15), were subsequently used to explore their effect on a stable fecal population via the fecal slurry model. For this purpose, we used a basal media composed of 2 g/l peptone water [Becton, Dickinson and Company (BD), Franklin Lakes, NJ, USA] 2 g/l yeast extract (BD), 0.1 g/l NaCl, 0.04 g/l K_2_HPO_4_, 0.04 g/l KH_2_PO_4_, 0.01 g/l MgSO_4_, 0.01 g/l CaCl_2_.⋅2H_2_O, 2 g/l NaHCO_3_, 2.5 g/l l-Cysteine-HCl, 0.5 g/l bile salts, 2 ml/l Tween-80, 1 g/l arabinogalactan, 2 g/l pectin, 1 g/l xylan, 4 g/l starch, 0.4 g/l glucose, and 0.4 g/l mucin type III (all purchased to Sigma-Aldrich). The mixture was homogenized and autoclaved for 15 min at 121°C, and the following components were added to the cooled media after sterilization by filtration (0.20 μm): 0.05 g/l bovine hemin (Sigma-Aldrich) and 10 μg/l vitamin K (Sigma-Aldrich). Before use, the basal media was maintained overnight at 37°C in anaerobiosis (10% v/v H_2_, 10%CO_2_, and 80% N_2_) in an anaerobic chamber Mac 500 (Don Whitley Scientific, West Yorkshire, UK).

#### Batch fecal slurry and sample points

The inoculum for the fecal slurry was prepared from the feces of a healthy adult volunteer (woman, 26 years old), who had not received antibiotics during the 6 months prior to the study. Feces were diluted (10% w/v) in sterile 0.17M phosphate buffered saline (pH 7) supplemented with 0.25% (w/v) cystein, and homogenized using a Lab-Blender 400 stomacher (Seward Medical, London, UK) for 2 min. Ten milliliters of the fecal homogenate were mixed with 90 ml of the basal media, and the fecal population was allowed to stabilize by an overnight incubation at 37°C in anaerobiosis.

Biopsy culture supernatants (500 μl) were added to 2 ml of the stabilized fecal slurry, and the mixes were incubated for 48 h at 37°C, including three tubes in which the same volume of un-conditioned media was added. Samples were collected by centrifugation (10 min, 16,000 × *g*, 4°C) at times 0 and 48 h. DNA isolation was performed using the QIAampDNA stool Mini kit (Qiagen, GmbH, Hilden, Germany) following the manufacturer instructions.

#### 16S rRNA gene profiling analysis

Partial 16S rRNA gene amplicons were obtained with primers Probio_Uni and/Probio_Rev (targeting the V3 and V4 region) by PCR (29). The products were purified, and a sequence library was prepared and sequenced in an Ion Torrent PGM system at the GenProbio Ltd., facilities^1^, using the Ion Sequencing 200 kit (Life Technologies, Thermo Fisher Scientific, Waltham, MA, USA). After sequencing, the individual sequence reads were filtered by the PGM software to remove low quality and polyclonal sequences. Sequences matching the PGM 3′ adaptor were also automatically trimmed. All PGM quality-approved, trimmed and filtered data were exported as.sff files.

The.sff files were processed using QIIME 1.7.0 running on an Ubuntu server (30). Sequences with a length between 150 and 200 bp and mean sequence quality score >25 were retained as part of our quality control. In addition, the sequence was trimmed at the first base if a low quality rolling 10 bp window was found. Presence of homopolymers >7 bp, and sequences with mismatched primers were omitted. In order to calculate downstream diversity measures (alpha and beta diversity indices, Unifrac analysis), 16S rRNA Operational Taxonomic Units (OTUs) were defined at ≥97% sequence homology. Chimeric sequences were removed using ChimeraSlayer. All reads were classified to the lowest possible taxonomic rank using QIIME and a reference dataset from GreenGenes^2^ (version 13.5, May 2013). OTUs were assigned using uclust (31) by using the script pick_de_novo_otus.py. The hierarchical clustering based on population profiles of most common and abundant taxa was performed using UPGMA clustering (Unweighted Pair Group Method with Arithmetic mean). This resulted in a Newick formatted tree, which was obtained utilizing the QIIME package. Alpha diversity was calculated through the alpha_diversity.py script using different metrics (Chao, Observed Species, Shannon and Simpson) to take into account the species evenness and richness.

The OTU tables were collapsed at five taxonomic levels (Phylum, Class, Order, Family, and Genus), exported in tab-delimited text format and analyzed using STAMP v2.0.1 (32). For each condition, the metagenomic profiles were evaluated at the different taxonomic levels. In each case, a non-supervised principal component analysis (PCA) was conducted, after which samples were classified in the different conditions: (i) absence or presence of biopsy supernatants in the fecal slurry and (ii) different biopsy/culture conditions. STAMP allows data filtering and analysis by the application of different statistical test’s and corrections. Association of taxa to the different grouping variables were identified by running two-sided Welch’s tests on every pair of means (two grouping variables) or ANOVA/Tukey Kramer (*post hoc*) tests (more than two grouping variables). The false discovery rate correction [FDR, (33)] was finally applied in all cases and significant differences in taxa were only considered below a *p*-value of 0.05 and a *q*-value below 0.2 (34).

#### Functional inference analysis

The functionality of the different metagenomes was predicted using the software PICRUSt 1.0.0^3^ (35). Briefly, this software allows the prediction of functional KEGG pathway abundances from the 16S rDNA reads. First, a collection of closed reference OTUs was obtained from the filtered reads using QIIME v1.7.0 (30) by querying the data against the GreenGenes database^2^ (version 13.5, May 2013). Reverse strand matching was enabled during the query and OTUs were picked at a 97 percent identity. A BIOM-formatted table (Biological Observation Matrix) was obtained with the pick_closed_reference_otus.py script. This table, containing the relative abundances of the different reference OTUs in all the metagenomes, was normalized by the predicted 16S rDNA copy number with the script normalize_by_copy_number.py. Final functional predictions, inferred from the metagenomes, were created with the script predict_metagenomes.py. When necessary, tab-delimited tables were obtained with the script convert_biom.py.

Predicted metagenomic contents were collapsed at the three hierarchical KEGG pathway levels^4^ with the categorize_by_function.py script. Each of these tables was analyzed statistically in STAMP v2.0.1 (32). Association of KEGG pathways at the different hierarchical levels with the different grouping variables were identified by running two-sided Welch’s tests on every pair of means (two grouping variables) or ANOVA/Tukey Kramer (*post hoc*) tests (more than two grouping variables). The FDR correction was finally applied in all cases and significant differences in KEGG pathways between groups were only considered below a *p*-value of 0.05 and a *q*-value below 0.2 (34). Data of the KEGG pathway distributions, at different hierarchical levels, were plotted with the script summarize_taxa_through_plots.py.

## Results

### Immune differences between the proximal and distal human colon

**Figure 1:**
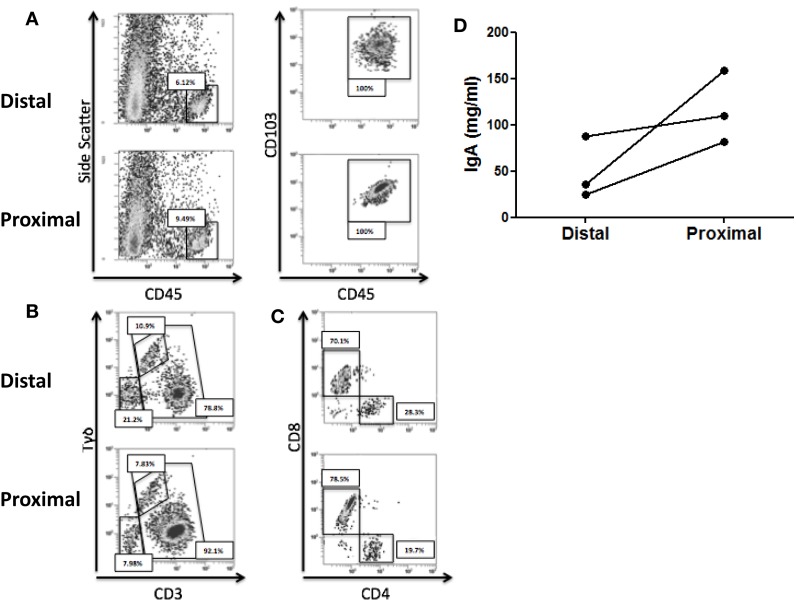
**Immune differences between the proximal and distal human colon**. **(A)** Intraepithelial lymphocytes (IEL) were identified, following DTT and EDTA treatment of the colonic biopsies as CD45^+^CD103^+^. **(B)** Total IELs were subsequently characterized as NK-like cells (CD3^−^), T-cells (CD3^+^), and Tγδ T-cells (CD3^+^γδ^+^). **(C)** Total T-cells (CD3^+^) within IEL were subsequently characterized for CD4 and CD8 expression. Results from **(A–C)** are representative of three independent experiments performed with similar results. **(D)** IgA content was determined on culture supernatants from paired proximal and distal colonic biopsies.

We first studied whether there were immune differences between the proximal and the distal colon. To that end, the IEL profile from each compartment was characterized for the three recruited healthy controls. Total IEL were identified by flow cytometry as CD45^+^ CD103^+^ and their numbers were higher in the proximal colon (Figure 1A). Further analysis revealed that the IEL compartment was constituted of NK-like cells (CD3^−^Tγδ^−^) and T-cells including both classical (CD3^+^Tγδ^−^) and Tγδ cells (CD3^+^Tγδ^+^) (36) (Figure 1B). All three controls had higher numbers of classical T-cells and lower numbers of Tγδ and NK-like cells in the proximal colon. Proportion of CD4/CD8 T-cell subsets revealed that the latter were higher in the proximal colon from the three studied controls (Figure 1C). We also studied the spontaneous production of soluble IgA elicited by the proximal and distal colon in basal culture supernatants, which could potentially modulate the microbiota composition and found that the proximal colon produced higher levels of IgA (Figure 1D). These results confirm the presence of immune differences between the proximal and distal colon of the three healthy volunteers.

### Fecal microbiota populations are modulated by the presence of colonic biopsies

When cultured in complete medium, colonic biopsies secrete soluble factors with modulatory effects on immune cells (37–39). Therefore, host-derived metabolites could also modulate and/or select the commensal microbiota. Having seen immune differences between the proximal and distal colon (Figure 1) we next studied whether colonic microenvironments from the compartments (i) had an effect on the gut microbiota and (ii) if that effect was differentially elicited by the proximal and distal colon. To that end, we used a fecal slurry model where a stabilized microbiota population was challenged with colonic culture supernatants from both proximal and distal colonic culture. As GIT inflammatory responses can modulate the microbiota composition (23), microbiota conditioning experiments were also performed after immune challenges of the colonic biopsies with pro-inflammatory innate cytokines (IL-15) or microbiota antigens (LPS). Negative controls included basal controls without any stimulation. Stabilized fecal microbiota was mixed with growth media conditioned by the different biopsies and incubated for 48 h. A fragment from the 16S rRNA gene was amplified by PCR and sequenced following a microbiota profiling approach as described in the material and methods section. This produced 18 microbial profiles based on the analysis of the 16S rRNA gene corresponding to the different experimental conditions [three individuals, two distal/proximal colon biopsies, three treatments (LPS-, IL-15-treated, or untreated)], two microbial profiles corresponding to the initial and final status of the fecal slurry batch, and three extra profiles in which un-conditioned media wash added to the fecal slurries.

**Figure 2:**
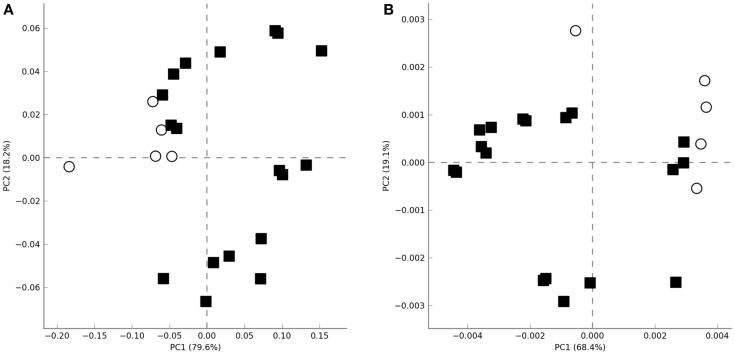
**Principal analysis component plots obtained using the total microbial population diversity (A) or the relative metabolic pathway abundances [(B), KEGG pathways] after functional inference through PICRUSt**. Axes represent the two linear variables containing the higher amount of variability. Open circles and closed squares represent samples were biopsia supernatants were absent or present, respectively.

Non-supervised PCA failed to cluster the microbial profiles according to the treatment (untreated, LPS, IL-15) or according to the biopsy location (proximal vs. distal colon). However, all microbiotas that were not exposed to colonic microenviroments [samples coming from the initial and final untreated fecal slurry batch (*t* = 0, *t* = 48) and those where only culture media had been added during incubation (*t* = 48)] clustered apart from the others where the biopsy supernatant had been present (Figure 2A). The same clustering was observed when metagenomic functionality was inferred from the metagenomes through PICRUSt pipeline (Figure 2B). This clustering was in agreement with the normalized microbiota composition, calculated in percentages at different taxonomic levels, which was different from microbiotas where the biopsy supernatant was present, compared with those where it was absent (Figure S1 in Supplementary Material).

**Figure 3:**
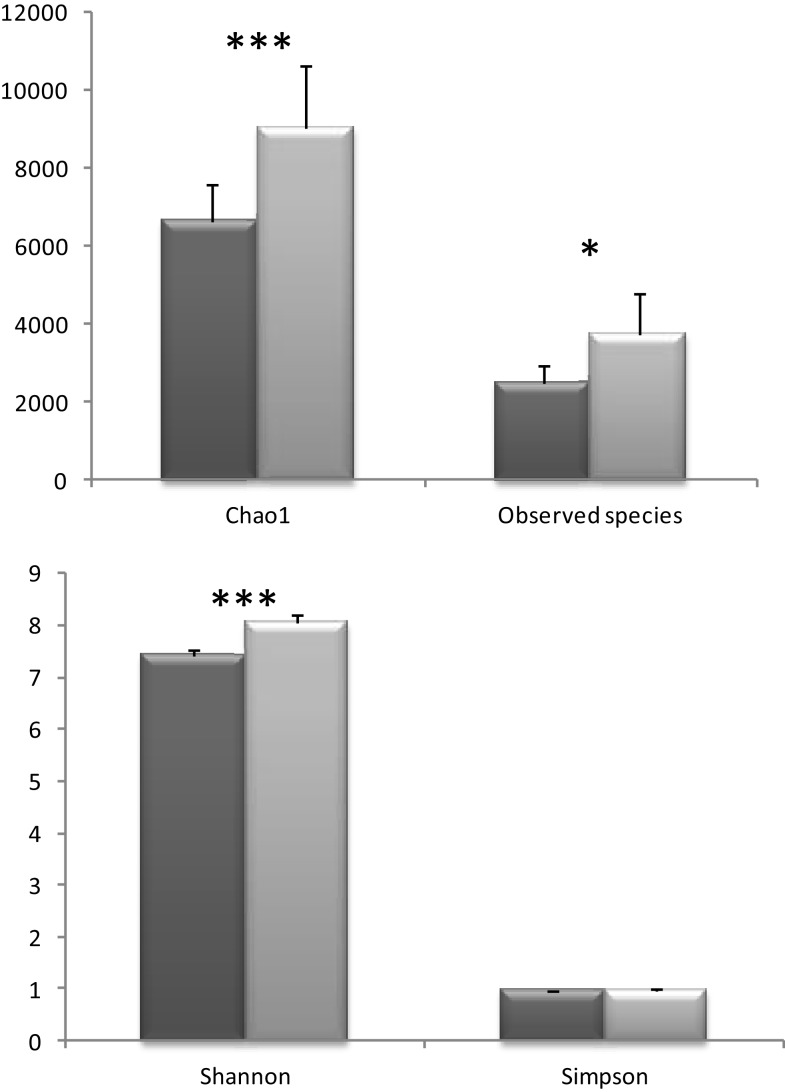
**Different alpha diversity indices in the samples were the colonic biopsia supernatants were absent (dark gray, basal) or present (light gray, biopsia)**. Bars represent the mean ± SD (*p < 0.05; ***p < 0.001).

When metagenomic data were clustered according to the presence or absence of a conditioned biopsy culture supernatant on the fecal slurry, an increase on the alpha diversity was noticed in the presence of the biopsy supernatant, whenever the index used was taking into account species richness (Chao 1, Observed Species) or evenness (Shannon) (no clear differences were found using Simpson index) (Figure 3). Species richness and evenness are two main components of species diversity. Whereas species richness is the number of species present in a sample, species evenness refers to the relative abundance of species.

### Compounds released by the colonic biopsies affect specific fecal microbiota populations

A more detailed analysis of the microbial groups showing changes in their populations, as affected by the presence or absence of host secreted compounds, revealed interesting trends in some microbial taxa (Table S1 in Supplementary Material). The Family *Prevotellaceae* (*q* < 0.12), *Enterococcaceae* (*q* < 0.08) and an unclassified Family belonging to the *Bacteroidales* Order (*q* < 0.09) showed moderate increases in the biopsy group. On the contrary, an unclassified family belonging to the β-Proteobacteria class showed lower relative abundance (*q* < 2E-04) when the biopsy-released compounds were present in the fecal slurry batch. Remarkably, several groups of microorganisms were only detectable in the presence of the biopsy supernatants (in at least the 50% of the samples). This included the Class TM7-3 (*q* < 0.03), the families *Methanobacteriaceae* (*q* < 0.05), *Victivallaceae* (*q* < 0.04), *Actinomycetaceae* (*q* < 0.18), *Turicibacteraceae* (*q* < 0.11), and *Eubacteriaceae* (*q* < 0.08), as well as an unclassified family belonging to the Order *Lactobacillales* (*q* < 0.11).

Using the same approach, we studied the effect of the biopsy supernatant on microbiota functionality by inferring information on relative KEGG pathway abundances using the PICRUSt software, as described in the material and methods section (Table S2 in Supplementary Material). When focused on the KEGG level #3, three pathways showed a statistically significant decrease in the presence of the biopsy. These pathways were Bacterial Chemotaxis (*q* < 1.87E-06), Two-component System (*q* < 0.01) and Glycerophospholipid Metabolism (*q* < 2.11E-06). On the contrary, several pathways showed significant increases, such as streptomycin biosynthesis (*q* < 4.56E-09), starch and sucrose metabolism (*q* < 0.17), ether lipid metabolism (*q* < 0.12), and fatty acid metabolism (*q* < 2E-03).

### Cytokine levels did not explain the observed differences in microbiota composition

The human colon produced soluble factors, which modulated both the fecal microbiota composition and its functionality in terms of KEGG pathway abundance as revealed by the fecal slurries batch experiments. However, there was no differential effect on microbiota composition elicited by the proximal or the distal colon, either in resting conditions or after IL-15 or LPS immune challenges. As human intestinal biopsies secrete several soluble immunomodulatory factors (37–39), we next determined the concentration of several cytokines and adipokines in the culture supernatants to correlate their concentrations with their observed effects on the microbiota. There was a high degree of inter-donor variability in terms of soluble cytokines concentration (Table 1), with the proximal colon secreting higher amounts of soluble IL-23, IL-27, and leptin (Figure S2 in Supplementary Material). Following culture, IL-15 *in vitro* challenge did not have any effect on the production of soluble immune mediators, although LPS increased the production of several cytokines (Figure S2 in Supplementary Material). Nevertheless, none of the determined soluble mediators correlated with the changes elicited on the microbiota composition and/or functionality after conditioning. Further analysis did not find a correlation between the observed microbiota modulation by the biopsy supernatants and either their IgA content or the IEL profile of the samples. Therefore, despite the fact that the human colonic microenvironment has the capacity to modulate the microbiota composition via soluble factors, such effect does not seem to be elicited by any of the measured immune mediators.

**Table 1:** **Cytokine levels on the biopsy supernatants**.

	Distal colon			Proximal colon		
	Basal	IL-15	LPS	Basal	IL-15	LPS
IL-6	146.4 ± 116.2	46.7 ± 34.7	280.1 ± 225.8	115.2 ± 90.7	349.5 ± 282.0	153.0 ± 117.5
IL-22	537.0 ± 418.6	6270.7 ± 322.6	1293.2 ± 742.7	487.5 ± 394.8	1424.2 ± 1029.1	858.6 ± 556.7
IL-9	10.9 ± 7.9	6.2 ± 4.1	30.3 ± 23.7	8.9 ± 6.3	28.6 ± 22.4	15.9 ± 12.0
IL-10	142.8 ± 114.2	93.6 ± 65.9	313.2 ± 253.4	114.6 ± 91.2	396.6 ± 321.4	212.7 ± 171.3
IL-27	51.1 ± 17.1	75.7 ± 18.8	73.9 ± 24.4	133.9 ± 91.6	137.0 ± 102.1	103.8 ± 42.0
IL-2	132.4 ± 107.7	93.1 ± 75.7	265.8 ± 216.7	143.3 ± 90.0	348.1 ± 283.4	204.5 ± 166.7
IL-17A	65.7 ± 52.9	41.9 ± 31.1	130.58 ± 105.8	54.9 ± 47.7	143.9 ± 113.5	106.3 ± 74.1
IFNg	124.6 ± 99.0	84.3 ± 66.0	290.7 ± 234.6	101.2 ± 79.9	329.7 ± 266.4	206.7 ± 166.0
IL-12(p70)	131.4 ± 95.2	137.5 ± 67.8	228.8 ± 178.0	182.4 ± 143.8	388.6 ± 302.9	220.2 ± 143.9
Leptin	254.0 ± 79.7	318.6 ± 65.5	299.9 ± 94.0	500.2 ± 322.7	505.8 ± 315.4	386.7 ± 132.1
TNF alpha	81.4 ± 64.0	51.3 ± 39.3	161.7 ± 125.7	63.6 ± 49.4	216.6 ± 174.3	69.3 ± 54.1
IL-1beta	71.9 ± 57.2	64.8 ± 51.4	241.6 ± 195.8	88.5 ± 69.9	246.8 ± 200.1	154.3 ± 124.5
IL-5	113.9 ± 92.3	83.3 ± 67.4	261.9 ± 201.3	124.0 ± 100.6	302.1 ± 246.0	223.7 ± 182.0
IL-4	75.4 ± 58.8	66.0 ± 52.9	232.4 ± 185.3	85.5 ± 66.9	298.4 ± 235.0	227.5 ± 177.3
IL-13	69.1 ± 52.1	45.8 ± 35.3	205.1 ± 159.7	80.7 ± 64.7	304.4 ± 248.2	168.1 ± 130.2
IL-23	218.1 ± 61.6	345.9 ± 66.7	178.5 ± 41.5	515.6 ± 335.8	429.9 ± 287.4	383.0 ± 160.5

## Discussion

Latest research on microbe/host crosstalk has been focused on the modulation of host processes by the intestinal microbiota through soluble metabolites (20–22, 40) in the framework of intestinal disorders (5–8) both in humans and animal models (41). The results presented in this paper show that such dialog is reciprocal, as soluble compounds released by the host also modulated the composition and functionality of a stable fecal population even resulting in the detection of otherwise undetectable components. Despite human proximal and distal colon having different properties (24–28), they did not elicit any differential effect on the microbiota composition/functionality, either in basal conditions or after an innate (IL-15) or microbiota-derived (LPS) immune challenge. On the contrary, our findings confirmed that the human colon secretes soluble factors with the ability to modulate the composition and functionality of a stable fecal microbiota although the induced changes did not correlate with any of the many soluble mediators identified in the supernatants.

When cultured in complete medium, GIT biopsies secrete several soluble factors with immunomodulatory effects on immune cells (2, 37, 39). A limitation of our approach is that in this case necrotic factors can be release in the media, as the epithelia prefer to be cultivated with one side exposed to the air. We have used this model to study whether such colonic-produced soluble factors had any differential effect on a gut microbiota, via a fecal slurry batch model (42). Although we considered colonic microenvironments from the proximal and the distal colon, both in resting conditions and after immune challenges (IL-15 or LPS), the only factor, which allowed us to group the results, was the presence/absence of the human colonic microenvironments. Such findings suggest that the changes induced in the microbiota were independent of the complete medium components but, on the contrary, elicited by colonic-derived soluble factors. The presence of the colonic supernatants resulted in the detection of some groups otherwise undetectable, such as the family *Methanobacteriaceae*. Members of this family, belonging to the *Archaea Domain*, are mainly represented by *Methanobrevibacter smithii* (43). This is one of the most abundant species in the human GIT, and it is known that its detection is strongly underestimated in 16S rRNA gene profiling approaches, both as result of limits in the technique and in the cell-wall composition of Archaea (29, 44). These results were in agreement with the relative metabolic pathway abundances inferred using PICRUSt and KEGG. Therefore, increases in *Methanobacteriaceae* can be correlated with the ether lipid metabolism, a pathway involved in the biosynthesis of ether-type polar lipids in Archaea (45). On the contrary, increases in Clostridiales can be linked with the starch and sucrose metabolism, as members of this group are well known by their saccharolytic ability (46).

Nevertheless, we cannot exclude the possibility that the soluble compounds with capacity to modulate the microbiota composition and function in our fecal slurry model are not host-derived. Indeed, it might be possible that the soluble are from bacterial origin, more concisely those isolated together with the biopsy. In this sense, it is known that extracellular compounds secreted by bacteria to the surrounding milieu, such as extracellular proteins/peptides, exopolysaccharides, lipoteichoic acids, or short chain fatty acids are able to modify the immune host response (40). However, our results strongly suggest that modifications on microbiota profiles are co-culture dependent, and therefore a molecule/molecules secreted by the host mucosa could be affecting the relative abundances of the gut microbe populations.

Traditional approaches aiming to modulate GIT microbiota included the use of antibiotics and/or pre/pro/synbiotics combinations which although effective to some extent, often showed short-term success (10). Fecal transplants, on the contrary, are being revealed as a promising tool to modulate the GIT microbiota (11–14). Nevertheless, there is variability in the outcome of fecal transplants, as they are not effective in all the patients, which in part is due to the stability of the resident human microbiota (15). The human colon secreted soluble factors able to modulate a stabilized fecal microbiota. Major individual, location, or challenge effects were ruled out since the modulation was elicited irrespective of the donor, sample location (proximal/distal colon), or immune status (basal/challenge). This is in agreement with other immune effectors secreted constitutively by the human host such as breast-milk IgA (47).

Despite immune differences between the proximal and the distal colon (Figure 1), they did not induce any differential effect on microbiota composition. However, both ecological niches are quite similar, so we cannot exclude a differential effect elicited by other gut compartments (e.g., terminal ileum, duodenum…). Also, all the colon biopsies used in this work were obtained from healthy adults without known autoimmune diseases, malignancies or any sign of inflammatory disorders. However, the effect of the diet and/or ongoing inflammatory processes (even with no clinical manifestations) cannot be discounted from having a role on the microbial modulation. For instance, higher levels of the families *Prevotellaceae* and *Unclassified Bacteroidaceae*, both belonging to the phylum *Bacteroidetes*, were measured in those fecal slurry batches where the biopsy supernatants were present. Increases in *Bacteroidetes* numbers were observed in other gut-related diseases such as, for instance, Type-2 Diabetes or Crohn’s Disease (48, 49), and also in a lean compared with an obese population (50). Moreover, lower *Bacteroidetes* numbers in cesarean-delivered infants, has been associated to an increased risk of developing allergic disease through a reduced Th1 response during the first two years of life (51). As the tissue microenvironment in patients suffering from IBD (or other GIT diseases) is different from that in healthy controls (2, 37), and also dependent on the diet (52), we cannot exclude the possibility that host-derived soluble factors, which modulate the GIT microbiota may be altered or even masked by ongoing pro-inflammatory responses in these patients.

In summary, we have shown that the human colon has the capacity to modulate the composition of a stabilized fecal microbial population via host-derived soluble factors. Patients with GIT diseases, including IBD (53), irritable bowel syndrome (IBS) (54), celiac disease (55), or type-1 diabetes (56) have altered microbiota composition thought to be a consequence of the immune response. Moreover, chronic inflammation in the gut such as in the framework of IBD could be a risk for colorectal cancer. As the host has the capacity to modulate the microbiota, it could provide a differential ecological niche and/or selective pressure on the microbiota via soluble factors which could be responsible (at least partially) for selecting the altered microbiota observed in such patients. Future studies should result in identification of such factors, but should also consider larger cohorts of both controls and patients to study any differential effects of age, diet, sampled tissue and/or presence of disease on modulating the microbiota. Factors produced in the intestinal environment – by either the host or the microbiota – could also be considered in those strategies aimed at modulating the microbiota composition. For instance, production of the molecule 2-aminoacetophenone, which is secreted by *Pseudomonas aeruginosa*, promotes bacterial intestinal colonization of model organisms by various means (57). Further research will assess if host or microbiota-derived soluble factors differ in those patients reacting to microbiota modulation (e.g., fecal transplant).

## Conflict of Interest Statement

The authors declare that the research was conducted in the absence of any commercial or financial relationships that could be construed as a potential conflict of interest.

## Supplementary Material

The Supplementary Material for this article can be found online at http://journal.frontiersin.org/article/10.3389/fonc.2015.00086/abstract

Figure S1 **Microbial composition of the different samples used in this study at several taxonomic levels, represented as the relative abundances (%)**.

Figure S2 **Differential production of cytokines in left vs. right colon (Slide #1), or with the samples grouped by treatment (Slides #2-9; 1: Basal, 2: IL-15 or 3: LPS)**.

Table S1 **Microbial groups showing statistical changes after grouping the samples using the absence (group 0) or presence (group 1) of biopsia supernatant in the fecal cultures**.

Table S2 **KEGG functions showing statistical changes after grouping the samples using the absence (group 0) or presence (group 1) of biopsia supernatant in the fecal cultures**.

## Footnotes

^1^http://www.genprobio.com

^2^http://greengenes.secondgenome.com

^3^http://picrust.github.com

^4^http://www.genome.jp/kegg/pathway.html
