# Supplementary figures and images for: Human Colon-Derived Soluble Factors Modulate Gut Microbiota Composition
Source: Front Oncol. 2015 Apr 13;5:86. doi: 10.3389/fonc.2015.00086 (PMC4394693; doi:10.3389/fonc.2015.00086)

# Supplementary Figure 1

## Phylum

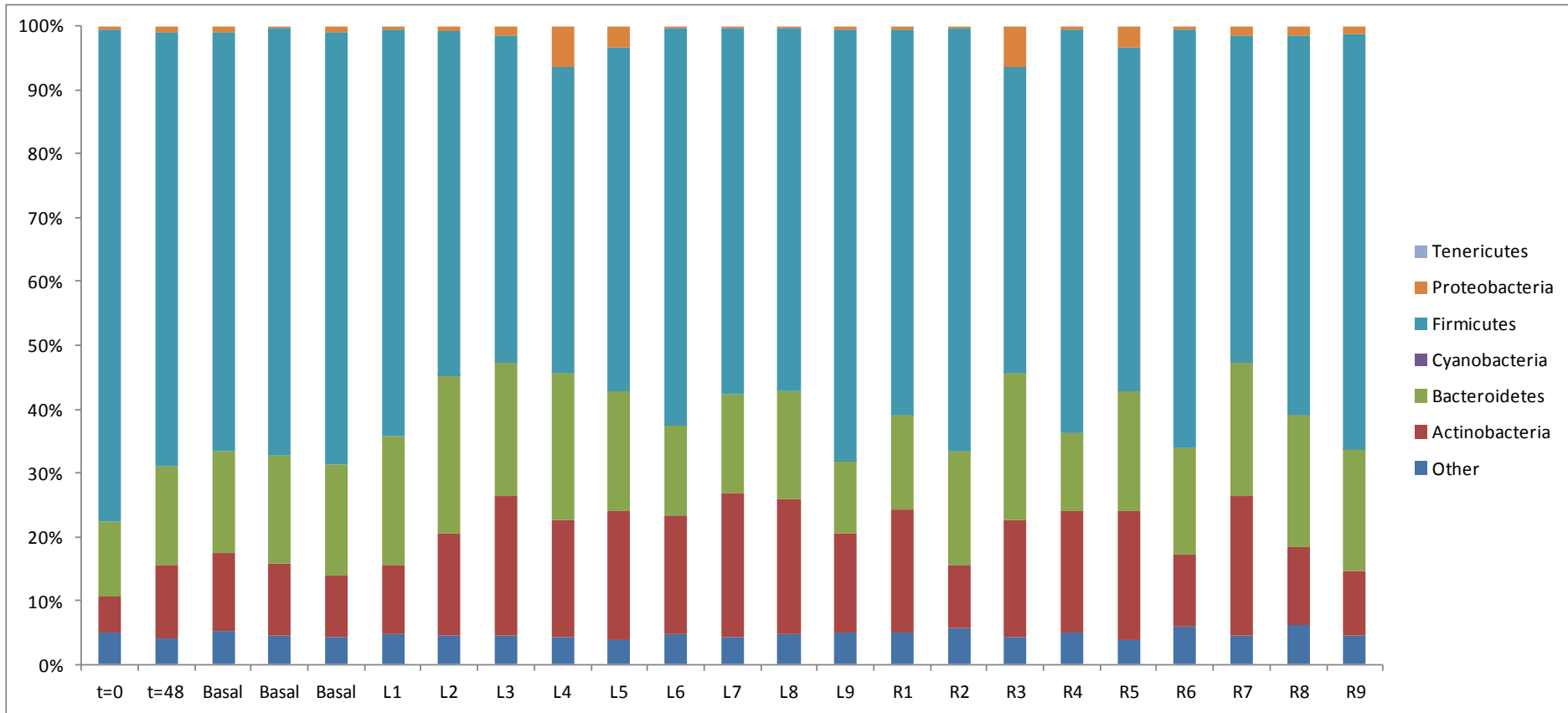

# Class

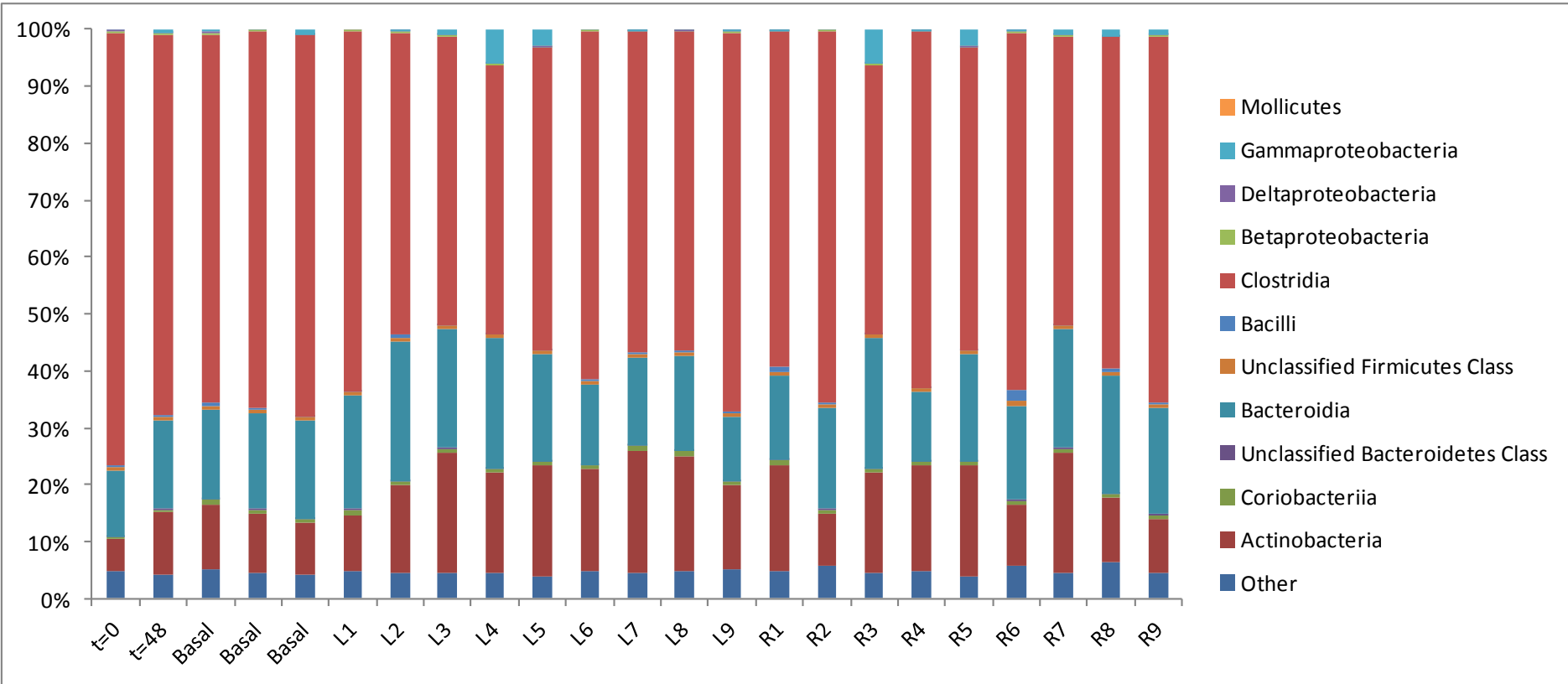

# Order

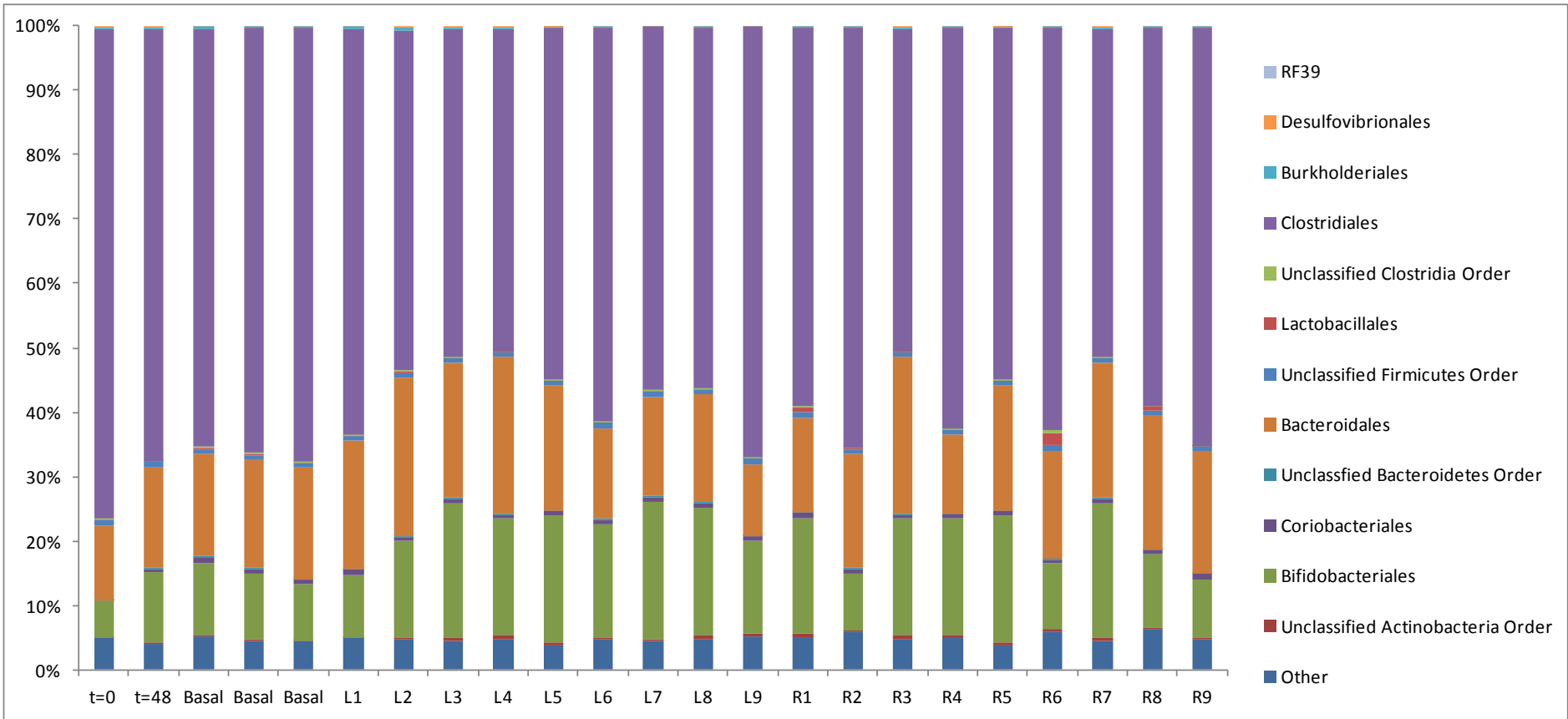

# Family

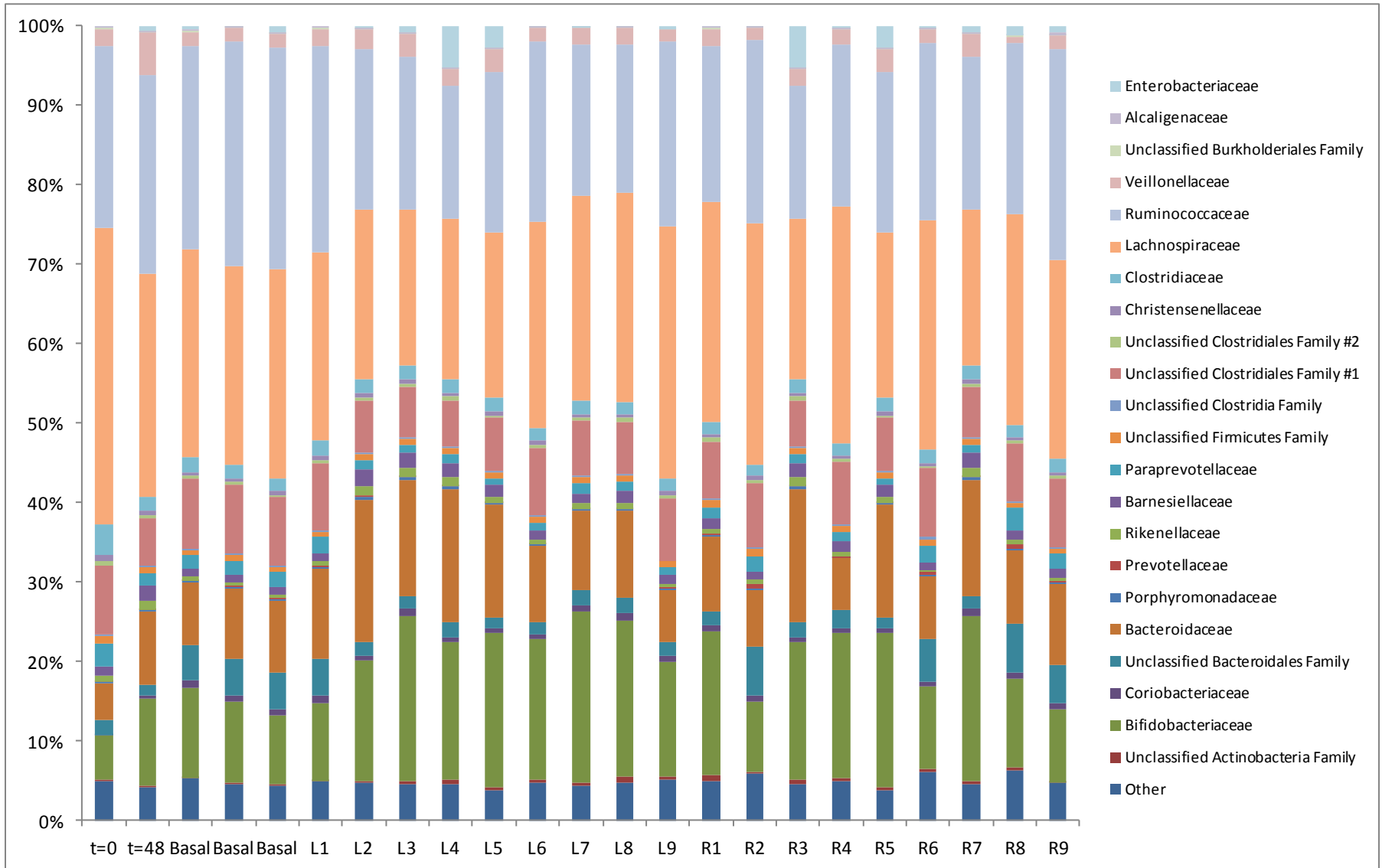

Supplement: Figure S1 — Microbial composition of the different samples used in this study at several taxonomic levels, represented as the relative abundances (%). [file Image_1.PDF]

# Supplementary Figure 2

Left vs right

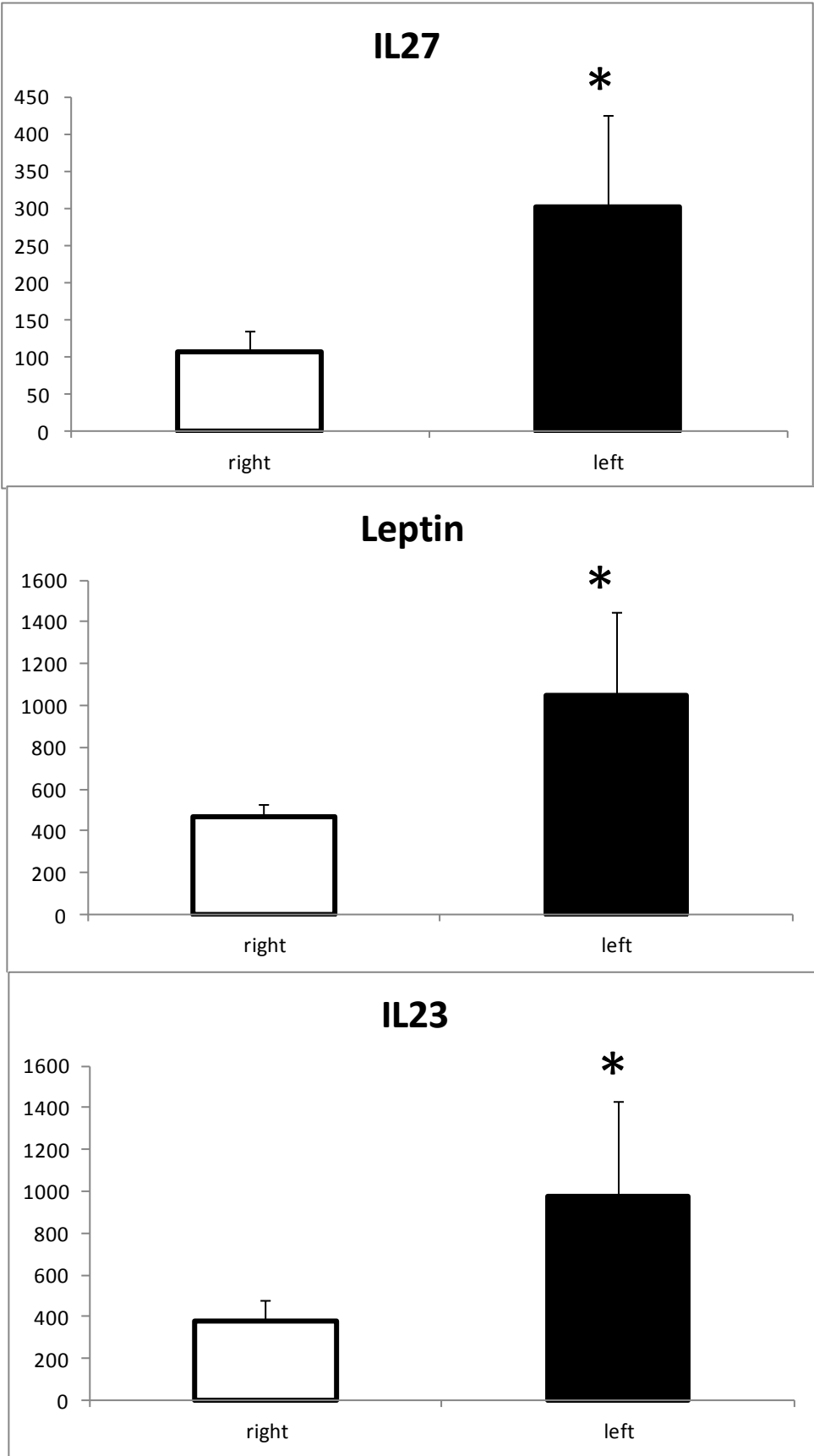

1: Basal, 2: IL-15, 3:LPS

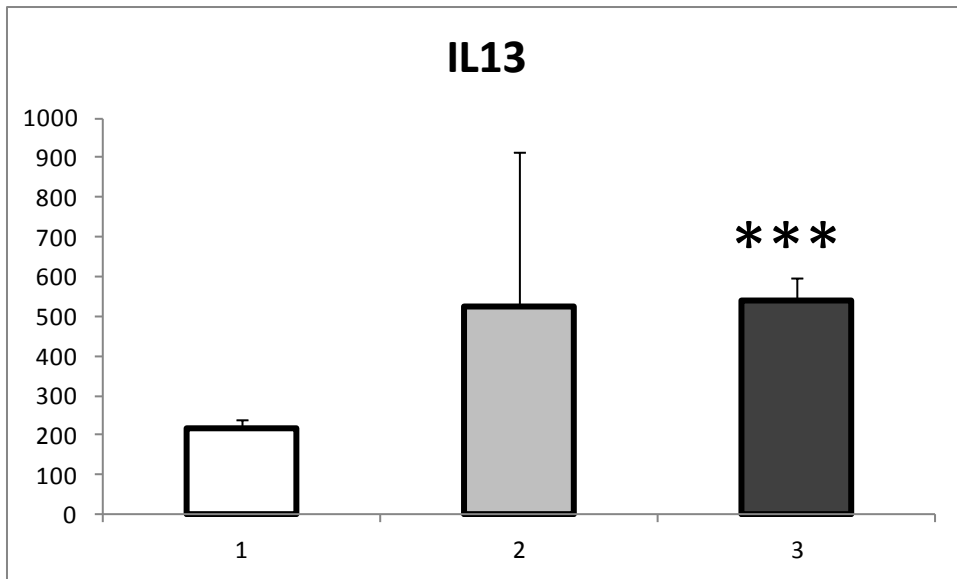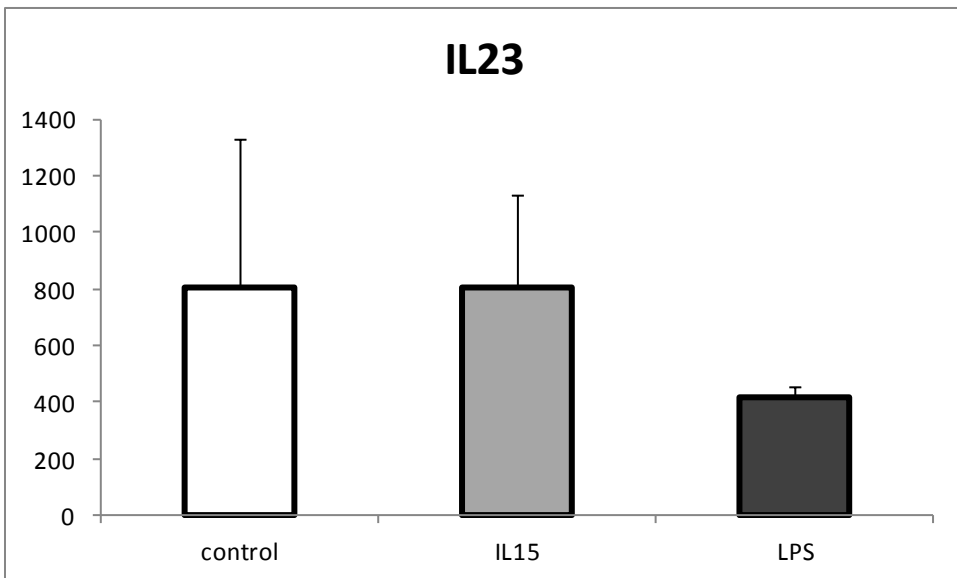

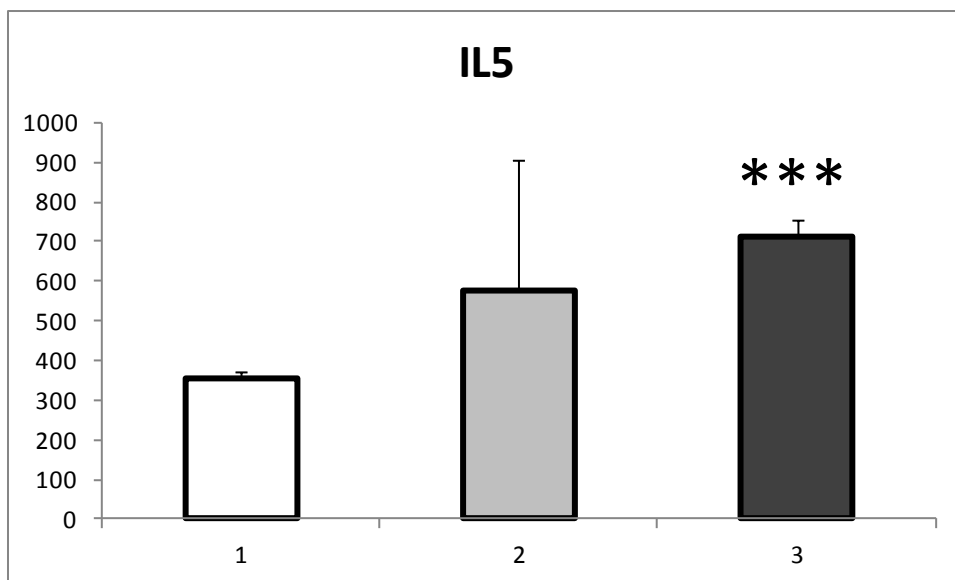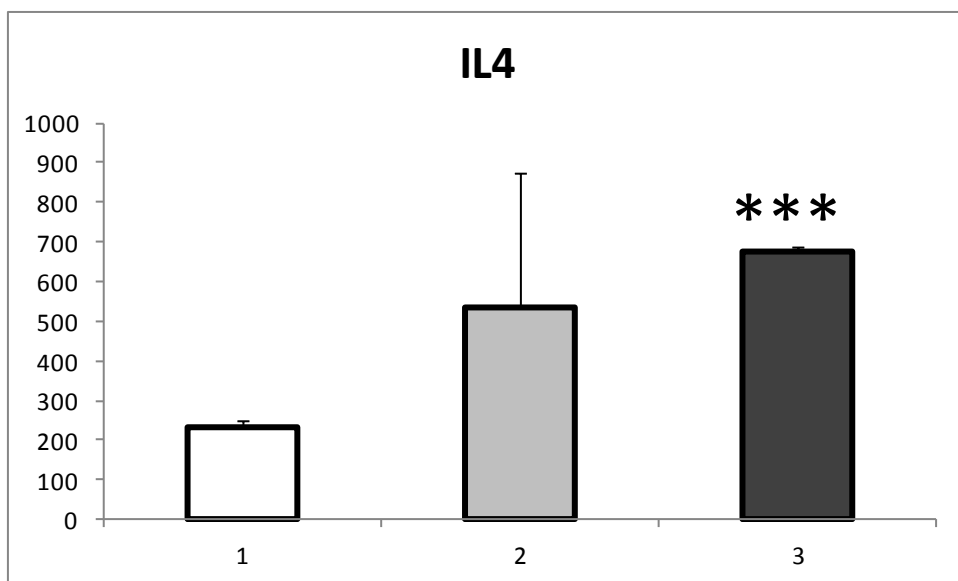

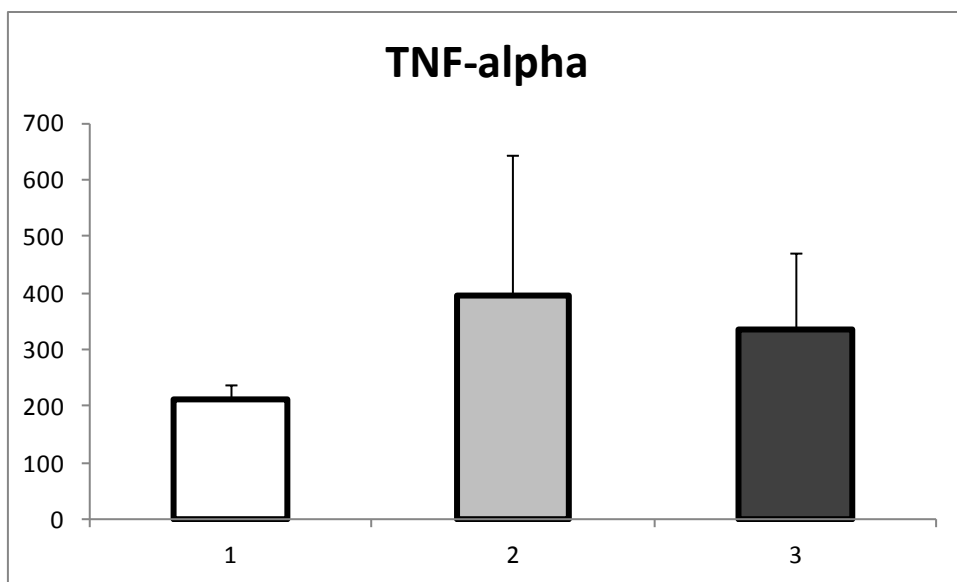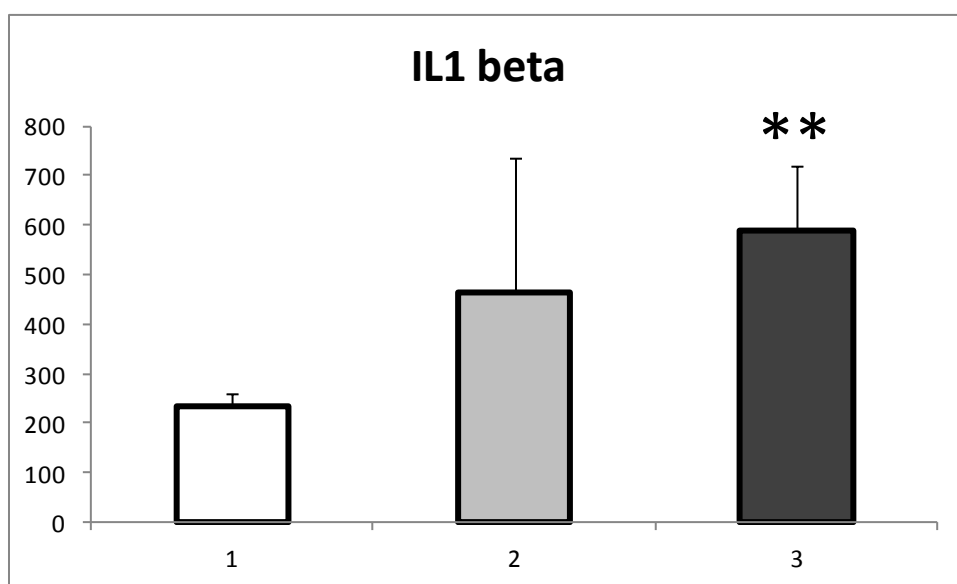

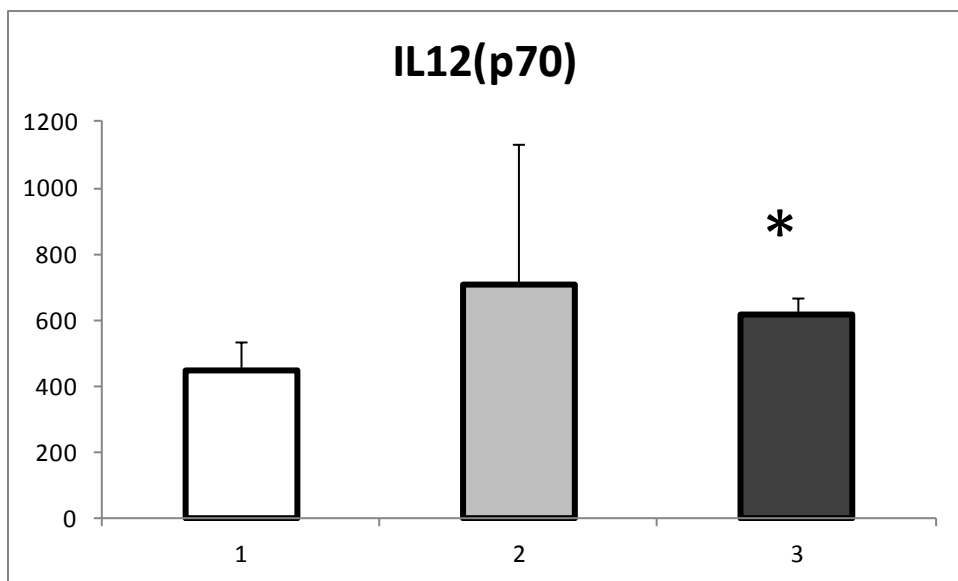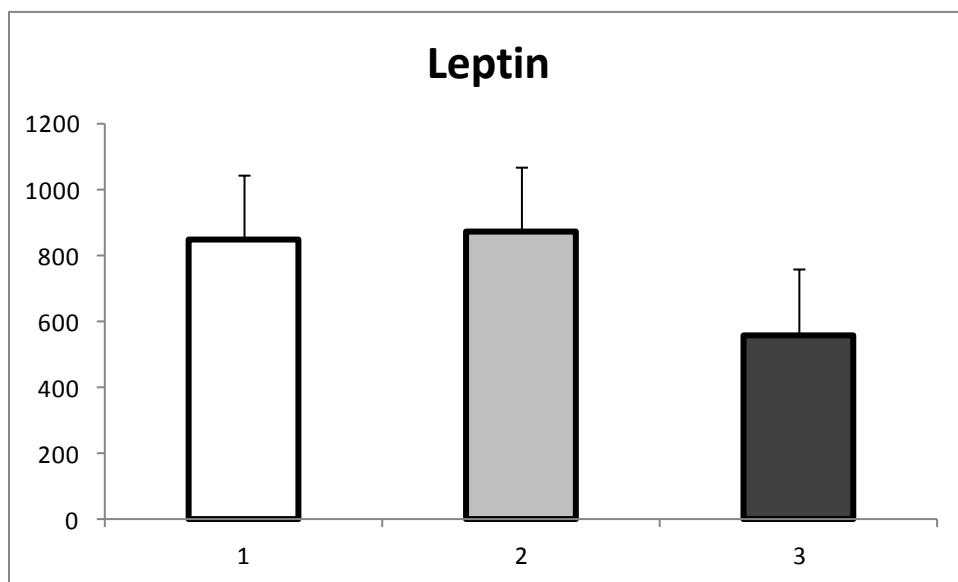

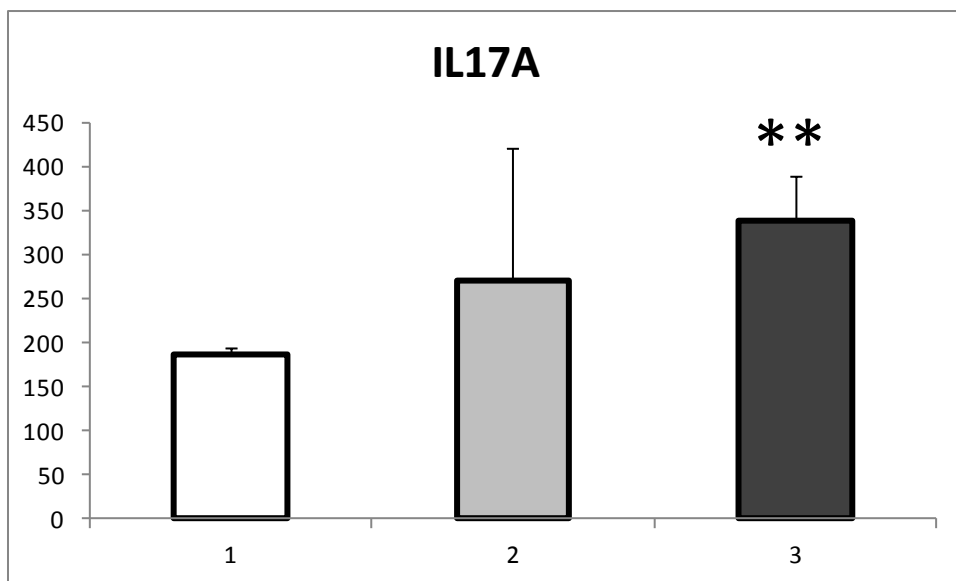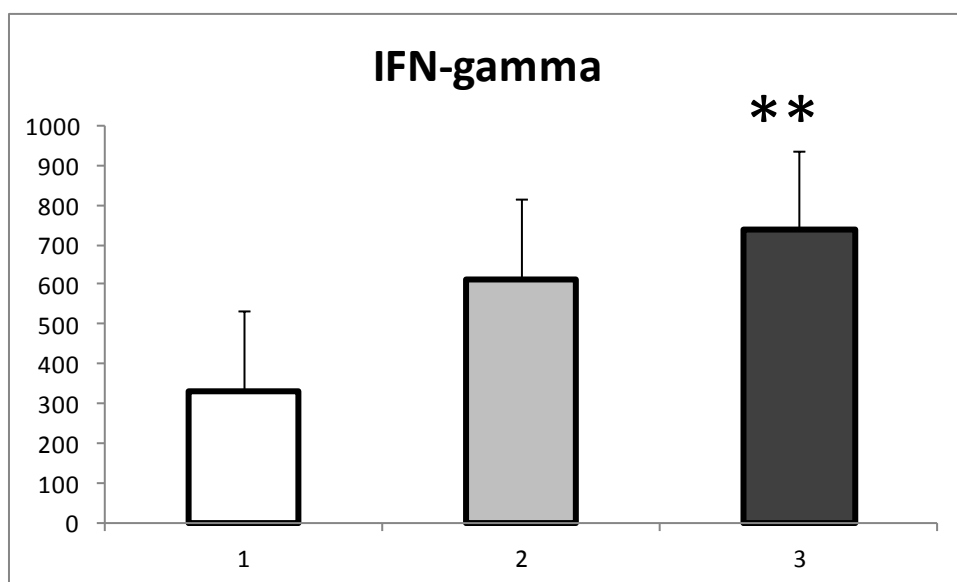

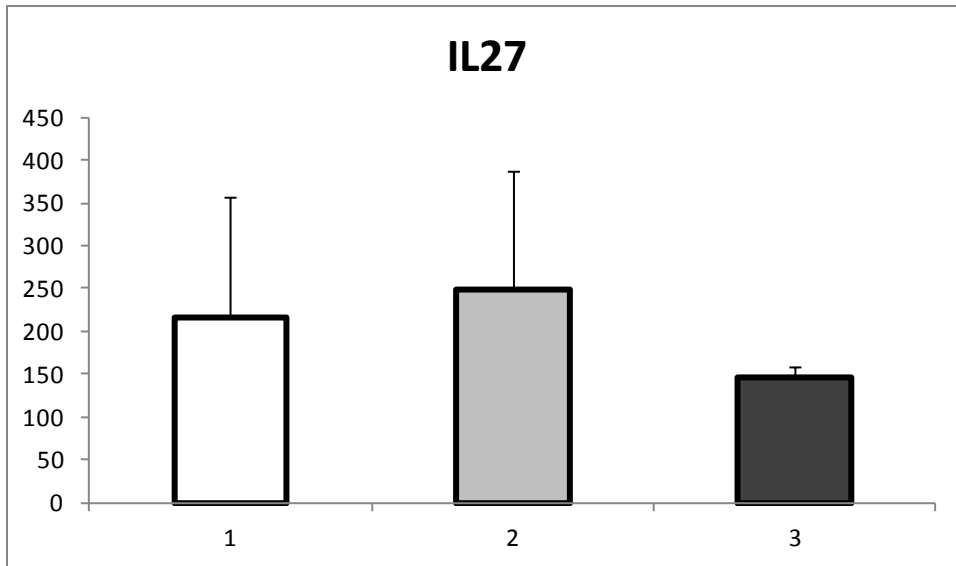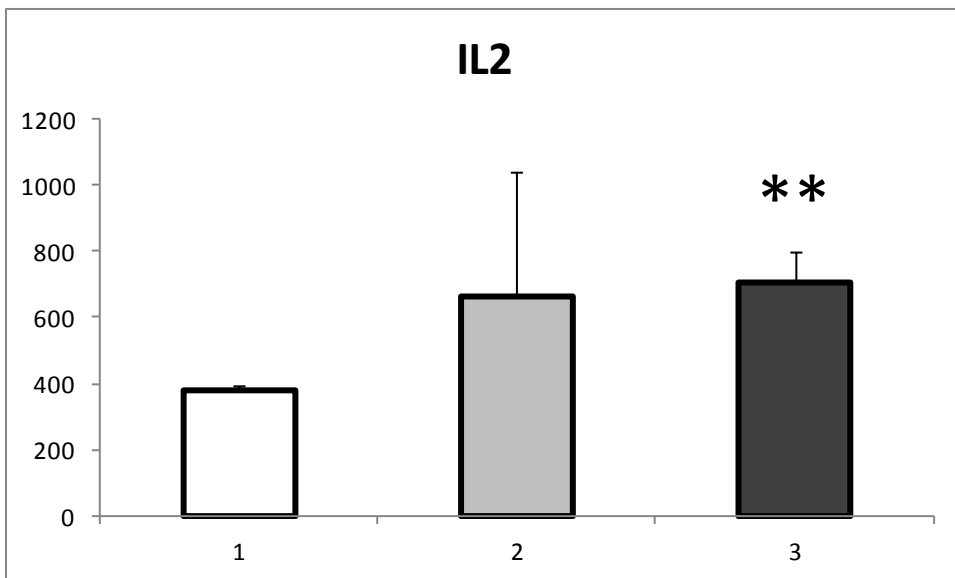

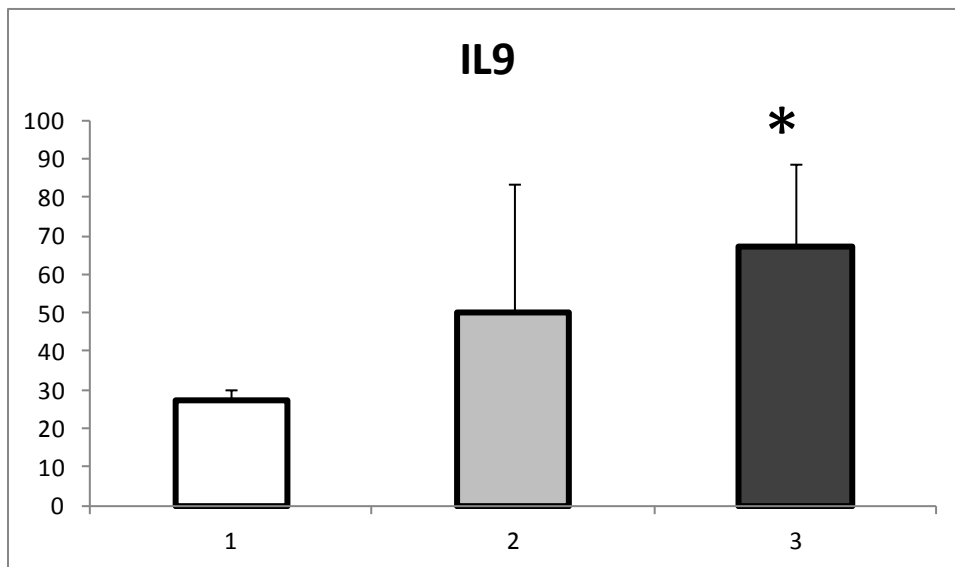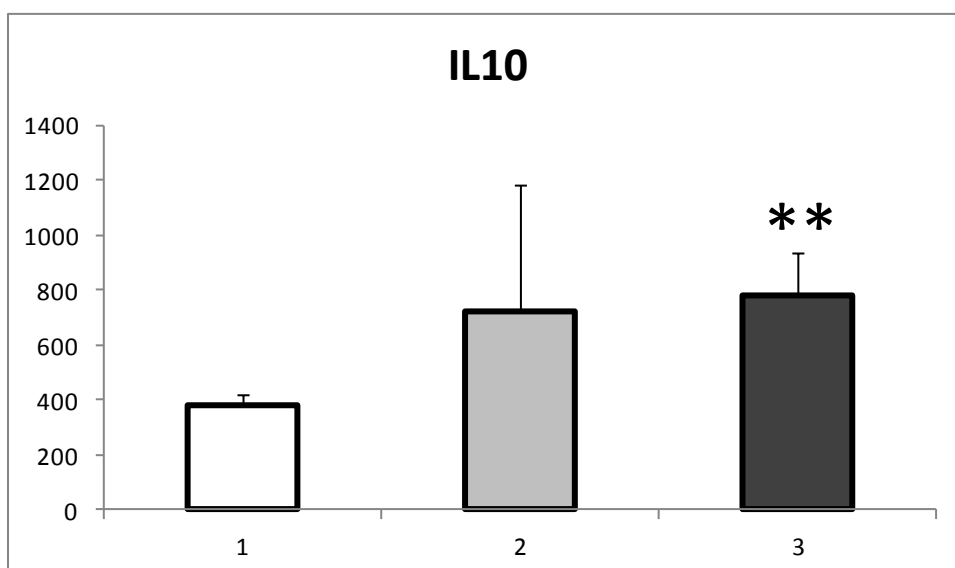

## IL6

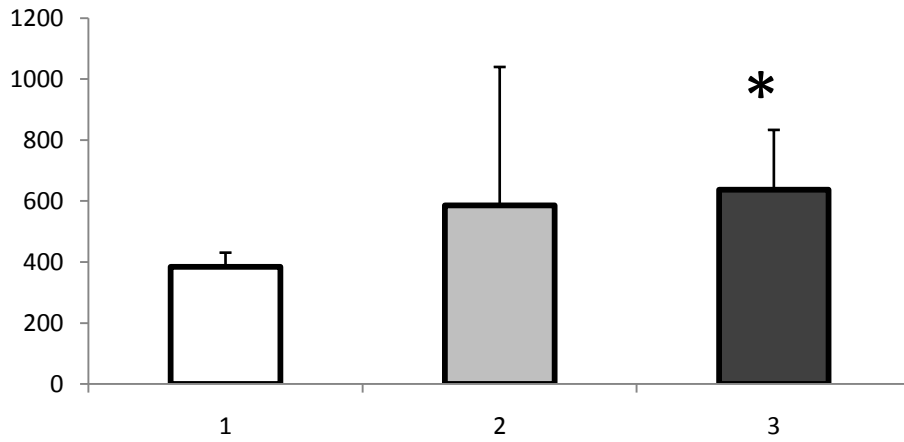

## IL22

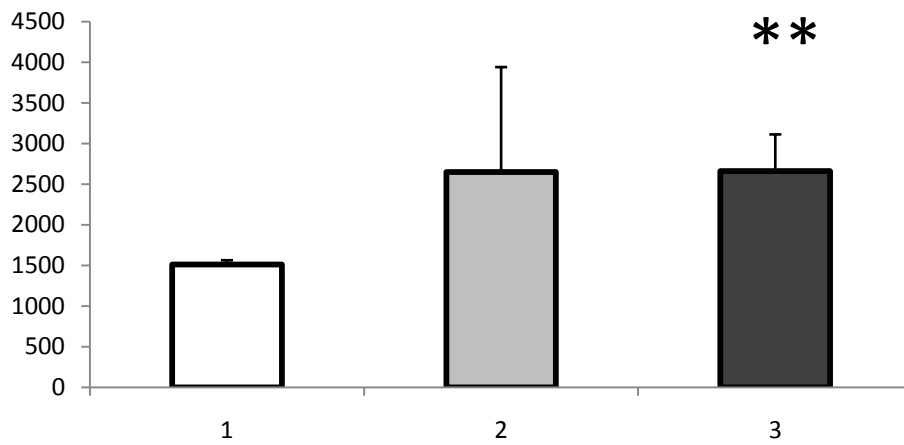

Supplement: Figure S2 — Differential production of cytokines in left vs. right colon (Slide #1), or with the samples grouped by treatment (Slides #2-9; 1: Basal, 2: IL-15 or 3: LPS). [file Image_2.PDF]
