# Supplementary material for: Human Colon-Derived Soluble Factors Modulate Gut Microbiota Composition
Source: Front Oncol. 2015 Apr 13;5:86. doi: 10.3389/fonc.2015.00086 (PMC4394693; doi:10.3389/fonc.2015.00086)
Supplement: Table S1 — Microbial groups showing statistical changes after grouping the samples using the absence (group 0) or presence (group 1) of biopsia supernatant in the fecal cultures. [file Table_1.PDF]

Supplementary Table 1

0: absence of biopsia	1: presence of biopsia

PHYLUM						
Taxon	0: mean rel. freq. (%)	0: std. dev. (%)	1: mean rel. f	1: std. dev. (%	p-values	q-values (corrected)
k__Archaea;p__Euryarchaeota	0.0	0.0	0.003237560	0.003650294	0.0019521907005	0.0253784791065
k__Bacteria;p__Lentisphaerae	0.0	0.0	0.003995936	0.004566039	0.00216943625342	0.00940089043147
k__Bacteria;p__TM7	0.0	0.0	0.001447833	0.002217090	0.0154136295458	0.0500942960237
CLASS						
Taxon	0: mean rel. freq. (%)	0: std. dev. (%)	1: mean rel. f	1: std. dev. (%	p-values	q-values (corrected)
k__Archaea;p__Euryarchaeota;c__Methanobacteria	0.0	0.0	0.003237560	0.003650294	0.0019521907005	0.0019521907005
k__Bacteria;p__Actinobacteria;Other	0.022948174957	0.0135248805732	0.078587361	0.019021721	0.120966483205	0.120966483205
k__Bacteria;p__Firmicutes;c__Bacilli	0.0840470732004	0.0384557537876	0.296278229	0.448761490	0.083514850324	0.083514850324
k__Bacteria;p__Firmicutes;Other	0.874944763702	0.0551181523189	0.732379125	0.086569547	0.199556278769	0.199556278769
k__Bacteria;p__Lentisphaerae;c__[Lentisphaeria]	0.0	0.0	0.003995936	0.004566039	0.00216943625341	0.00216943625341
k__Bacteria;p__Proteobacteria;c__Deltaproteobacteria	0.111249349598	0.00183018300701	0.084437795	0.051328515	0.0474757347528	0.0474757347528
k__Bacteria;p__Proteobacteria;c__Epsilonproteobacteria	0.0	0.0	0.000909422	0.002342337	0.127835894082	0.127835894082
k__Bacteria;p__Proteobacteria;Other	0.0	0.0	0.008331090	0.008618757	0.000956823179546	0.000956823179546
k__Bacteria;p__TM7;c__TM7-3	0.0	0.0	0.001158843	0.002040959	0.0316728723732	0.0316728723732
ORDER						
Taxon	0: mean rel. freq. (%)	0: std. dev. (%)	1: mean rel. f	1: std. dev. (%	p-values	q-values (corrected)
k__Archaea;p__Euryarchaeota;c__Methanobacteria;o__Methanobacteriales	0.0	0.0	0.003237560	0.003650294	0.0019521907005	0.027330669807
k__Bacteria;p__Firmicutes;c__Bacilli;o__Turicibacterales	0.0	0.0	0.002550900	0.003739546	0.0119847214348	0.111857400058
k__Bacteria;p__Lentisphaerae;c__[Lentisphaeria];o__Victivallales	0.0	0.0	0.003995936	0.004566039	0.00216943625341	0.0242976860382
k__Bacteria;p__Proteobacteria;c__Betaproteobacteria;Other	0.00927077913314	0.000152515250582	0.002904677	0.003695165	1.67368586168e-06	9.37264082541e-05
k__Bacteria;p__Proteobacteria;Other;Other	0.0	0.0	0.008331090	0.008618757	0.000956823179544	0.0267910490272
FAMILY						
Taxon	0: mean rel. freq. (%)	0: std. dev. (%)	1: mean rel. f	1: std. dev. (%	p-values	q-values (corrected)
k__Archaea;p__Euryarchaeota;c__Methanobacteria;o__Methanobacteriales;f__Methanobacteriaceae	0.0	0.0	0.003237560	0.003650294	0.0019521907005	0.0463645291369
k__Bacteria;p__Actinobacteria;c__Actinobacteria;o__Actinomycetales;f__Actinomycetaceae	0.0	0.0	0.001801787	0.002961460	0.0225497600004	0.178518933336
k__Bacteria;p__Bacteroidetes;c__Bacteroidia;o__Bacteroidales;f__Prevotellaceae	0.0373881470337	0.0191516192686	0.159310637	0.147777205	0.0112711044453	0.118972769144
k__Bacteria;p__Bacteroidetes;c__Bacteroidia;o__Bacteroidales;Other	1.52877979104	0.25222284748	3.198808019	1.675306548	0.00652062793635	0.088494236279
k__Bacteria;p__Firmicutes;c__Bacilli;o__Lactobacillales;f__Enterococcaceae	0.00927077913314	0.000152515250578	0.018009551	0.011710629	0.00686709500428	0.0815467531759
k__Bacteria;p__Firmicutes;c__Bacilli;o__Lactobacillales;Other	0.0	0.0	0.002808955	0.004131454	0.0122207558173	0.105542891149
k__Bacteria;p__Firmicutes;c__Bacilli;o__Turicibacterales;f__Turicibacteraceae	0.0	0.0	0.002550900	0.003739546	0.0119847214347	0.11385485363
k__Bacteria;p__Firmicutes;c__Clostridia;o__Clostridiales;f__Eubacteriaceae	0.0	0.0	0.001577241	0.002028210	0.0051764426494	0.0819603419489
k__Bacteria;p__Lentisphaerae;c__[Lentisphaeria];o__Victivallales;f__Victivallaceae	0.0	0.0	0.003995936	0.004566039	0.00216943625341	0.0412192888148
k__Bacteria;p__Proteobacteria;c__Betaproteobacteria;Other;Other	0.00927077913314	0.000152515250578	0.002904677	0.003695165	1.67368586146e-06	0.000159000156839
k__Bacteria;p__Proteobacteria;Other;Other;Other	0.0	0.0	0.008331090	0.008618757	0.000956823179545	0.0454491010284
