# Supplementary material for: Human Colon-Derived Soluble Factors Modulate Gut Microbiota Composition
Source: Front Oncol. 2015 Apr 13;5:86. doi: 10.3389/fonc.2015.00086 (PMC4394693; doi:10.3389/fonc.2015.00086)
Supplement: Table S2 — KEGG functions showing statistical changes after grouping the samples using the absence (group 0) or presence (group 1) of biopsia supernatant in the fecal cultures. [file Table_2.PDF]

# Supplementary Table 2

0= absence of biopsia 1= presence of biopsia

KEGG Level 1

| Taxon | 0: mean rel. freq. (%) | 0: std. dev. (%) | 1: mean rel. freq. (%) | 1: std. dev. (%) | p-values | q-values (corrected) |
|---|---|---|---|---|---|---|
| Cellular Processes | 3.07254798923 | 0.0386337487098 | 2.72556779553 | 0.0956609901579 | 0.0207976777796 | 0.0831907111183 |
| Metabolism | 46.7526077615 | 0.0346911910427 | 47.2519604393 | 0.198033504522 | 6.54591581591e-05 | 0.000523673265273 |

KEGG Level 2

| Taxon | 0: mean rel. freq. (%) | 0: std. dev. (%) | 1: mean rel. freq. (%) | 1: std. dev. (%) | p-values | q-values (corrected) |
|---|---|---|---|---|---|---|
| Environmental Information Processing;Signal Transduction | 1.5432006075 | 0.010825588938 | 1.46407718298 | 0.0374300676801 | 0.0132566390263 | 0.181174066693 |
| Unclassified;Metabolism | 2.32458377125 | 0.000994947492192 | 2.34875524771 | 0.0212250897497 | 0.00022117603856 | 0.00906821758097 |
| Unclassified;Poorly Characterized | 4.66483425355 | 0.0020271352938 | 4.68683275084 | 0.023282268035 | 0.00192828192969 | 0.0395297795586 |

KEGG Level 3

| Taxon | 0: mean rel. freq. (%) | 0: std. dev. (%) | 1: mean rel. freq. (%) | 1: std. dev. (%) | p-values | q-values (corrected) |
|---|---|---|---|---|---|---|
| Cellular Processes;Cell Motility;Bacterial chemotaxis | 0.528193400266 | 0.00331118150261 | 0.437591643891 | 0.0320338648722 | 1.71479410671e-08 | 1.87484155667e-06 |
| Environmental Information Processing;Signal Transduction;Two-component system | 1.43139468403 | 0.00668059467911 | 1.34334241055 | 0.035342809124 | 0.000183254230564 | 0.00858676966071 |
| Metabolism;Biosynthesis of Other Secondary Metabolites;Streptomycin biosynthesis | 0.323184013909 | 0.00026878881146 | 0.338370901224 | 0.00402740793686 | 1.39010030913e-11 | 4.55952901396e-09 |
| Metabolism;Carbohydrate Metabolism;Starch and sucrose metabolism | 1.19355933087 | 0.00344494858868 | 1.21996302387 | 0.0137998824429 | 0.00726990795195 | 0.170323557731 |
| Metabolism;Energy Metabolism;Photosynthesis - antenna proteins | 0.0 | 0.0 | 5.1115342628e-05 | 6.17785196671e-05 | 0.00332440428832 | 0.109040460657 |
| Metabolism;Lipid Metabolism;Ether lipid metabolism | 0.000804102288604 | 2.71329218035e-05 | 0.00101559829966 | 0.000126441359038 | 0.00417941802469 | 0.124622646554 |
| Metabolism;Lipid Metabolism;Fatty acid metabolism | 0.215810571833 | 0.00114181808365 | 0.231496139918 | 0.00913096368272 | 3.4597653588e-05 | 0.00283700759422 |
| Metabolism;Lipid Metabolism;Glycerophospholipid metabolism | 0.582025218349 | 0.000210287722568 | 0.562977363262 | 0.00781790525011 | 1.28505281971e-08 | 2.10748662433e-06 |
